# Trends in Use of Prescribed Opioids in Incident and Prevalent Patients With Ulcerative Colitis: A Nationwide Study in Sweden

**DOI:** 10.1093/ibd/izaf278

**Published:** 2025-12-11

**Authors:** Mehdi Osooli, Siri Voghera, Gustaf Bruze, Caroline Nordenvall, Charlotte R H Hedin, Åsa H Everhov, Pär Myrelid, Jonas F Ludvigsson, Ola Olén, SWIBREG Study Group§, Malin Olsson, Henrik Hjortswang, Jonas Bengtsson, Hans Strid, Marie Andersson, Susanna Jäghult, Jonas Halfvarson, Martin Rejler, Olof Grip, Ulrika L Fagerberg, Karl Mårild, Johann Hreinsson, Pontus Karling

**Affiliations:** Division of Clinical Epidemiology, Department of Medicine Solna, Karolinska Institutet, Stockholm, Sweden; Division of Clinical Epidemiology, Department of Medicine Solna, Karolinska Institutet, Stockholm, Sweden; Division of Clinical Epidemiology, Department of Medicine Solna, Karolinska Institutet, Stockholm, Sweden; Department of Molecular Medicine and Surgery, Karolinska University Hospital, Stockholm, Sweden; Department of Pelvic Cancer, Unit of IBD-surgery, Karolinska University Hospital, Stockholm, Sweden; Karolinska University Hospital, Centre for Digestive Health, Department of Gastroenterology Dermatovenereology and Rheumatology, Karolinska University Hospital, Stockholm, Sweden; Department of Medicine Solna, Karolinska Institutet, Stockholm, Sweden; Division of Clinical Epidemiology, Department of Medicine Solna, Karolinska Institutet, Stockholm, Sweden; Department of Clinical Science and Education Södersjukhuset, Karolinska Institutet, Stockholm, Sweden; Department of Surgery and Department of Biomedical and Clinical Sciences, Linköping University, Linköping, Sweden; Department of Medical Epidemiology and Biostatistics, Karolinska Institutet, Stockholm, Sweden; Department of Pediatrics, Örebro University Hospital, Örebro, Sweden; Division of Digestive and Liver Disease, Department of Medicine, Columbia University Medical Center, New York, NY, United States; Division of Clinical Epidemiology, Department of Medicine Solna, Karolinska Institutet, Stockholm, Sweden; Sachs’ Children and Youth Hospital, Stockholm South General Hospital, Stockholm, Sweden

**Keywords:** inflammatory bowel disease, ulcerative colitis, diagnosis, opioids, prevalence, incidence

## Abstract

**Background and Aims:**

Opioids are not optimal for managing pain among patients with ulcerative colitis (UC), but the use of opiods in nationwide UC populations remains unexplored. We aimed to describe dispensed opioid use around UC diagnosis and annual trends among patients with prevalent UC.

**Methods:**

We performed a nationwide population-based cohort study of adults with an incident (2008-2020) or a prevalent (2006-2022) UC diagnosis and matched reference individuals from the general population. We obtained data on opioid dispensations and estimated the prevalence of having ≥1 dispensation per six-month period from two years before until five years following a first UC diagnosis. We also estimated annual opioid use among participants with a prevalent UC diagnosis and their matched reference individuals.

**Results:**

Overall, 66 929 adults with UC (including 25 417 patients with an incident diagnosis) and 641 609 matched reference individuals were included. Compared to reference individuals, patients with UC had a 1.3-fold higher prevalence of opioid use (6.4% vs 4.9%) two years before diagnosis, which peaked during the year of diagnosis (11.0%) and stabilized at a 1.7-fold higher use three to five years after diagnosis. Between 2006 and 2022 the annual prevalence of opioid use decreased by 15.0% in patients with UC and by 11.0% in reference individuals.

**Conclusion:**

In this nationwide register-based study, adults with UC had higher prescribed opioid use within two years before and up to five years after first UC diagnosis compared with reference individuals. However, adults with prevalent UC (and reference individuals) had a declining temporal opioid use trend during 2006-2022.

Key Messages *What is already known?*

The use of opioids for pain management among individuals with ulcerative colitis is not recommended and should be minimized due to limited evidence of benefit and potential for harm.

*What is new here?*

We found that the use of prescribed opioids among individuals with ulcerative colitis was already substantially higher than in the general population two years preceding diagnosis and that the use peaked the year of UC diagnosis and remained elevated for up to five years after diagnosis. Additionally, we observed 15% and 11% declines in annual prescribed opioid use among UC patients and the general population in Sweden between 2006 and 2022.

*How can this study help patient care?*

Despite a declining annual trend of opioid use among both adults with ulcerative colitis (UC) and the general population, prescribed opioids continue to be used for pain management in UC. Increasing awareness of the risks associated with opioids and the promotion of safer, evidence-based alternatives for pain management may help sustain and accelerate this downward trend.

## Introduction

Ulcerative colitis (UC) is a subtype of inflammatory bowel disease (IBD) with a range of debilitating symptoms, including abdominal pain, rectal bleeding, diarrhea, and urgency. Pharmacological therapies in UC patients primarily aim to induce and maintain clinical remission. However, in a considerable proportion of patients UC remains refractory to these treatments, necessitating colectomy.^1^ For many patients pain resolves with successful treatment of the inflammation, but for patients with refractory IBD persistent pain can be a challenging problem. In addition, pain may persist among patients with UC in remission.^2^

Opioids serve as effective analgesics for most UC-related abdominal pain but pose significant risks of harm, including dependence and adverse effects including opioid-related deaths. Physicians are advised to investigate the causes of pain, consider non-opioid alternatives, and provide psychological support.^3^ The United States declared opioid use a public health emergency in 2017, with 220 daily overdose deaths in 2021—nearly double the rate in 2010.^4^^,^^5^ About one-third of opioid-related deaths involve opiods prescribed to patients with opioid use disorder (OUD). Although Europe faces lower opioid-related mortality than the United States, some countries, like Sweden, report high opioid-related deaths.^6^

Evidence of the effectiveness of opioids in managing abdominal pain among adults with IBD (including UC and Crohn disease [CD]) is still scarce and inconsistent.^7^ In addition, the use of opioids among patients with IBD including UC have been associated with increased mortality,^8^^,^^9^ drug dependence,^10^ and higher burdens for the healthcare system, such as longer hospital stays,^11^ although some of the poor outcomes are a consequence of confounding by indication. Several studies have described opioid use among patients with UC; however, these studies have been limited by a cross-sectional design^12^ and are based on United States data^12–16^ and thus not necessarily generalizable to a European context. Other limitations are that some of the previous studies have been limited to larger referral centers, did not include a nationwide patient cohort and were limited to the selected populations of larger referral centers, or did not include a nationwide patient cohort.^8^^,^^15^ A list of previous studies and their characteristics is provided in Appendix Table S1.

Descriptive data on opioid use in IBD are essential for guiding both clinical decision-making and health policy. Such data illuminate prescribing patterns, highlight populations at elevated risk for adverse outcomes, and are useful for assessing the effectiveness of strategies aimed at minimizing opioid exposure. These findings also serve to inform the development of updated clinical guidelines and support the design of healthcare resource planning in chronic disease management.^8^

We therefore aimed to investigate the use of prescribed opioids among adults with UC in Sweden. We leveraged comprehensive national health registers to achieve the following aims: (1) to describe opioid use two years before and up to five years following the first diagnosis of UC and the corresponding date among matched reference individuals and (2) to explore the trend of annual opioid use among patients with a prevalent UC diagnosis exposed to modern IBD care compared with matched reference individuals during the period 2006-2022.

## Materials and Methods

### Study Design

In this population-based cohort study, we compared adults diagnosed with UC in Sweden and matched them reference individuals from the general population by using nationwide register data, which were prospectively collected from routine clinical practice.

### Setting

Sweden is a high-income country with 10.4 million residents as of December 2020.^17^ All Swedish residents have access to tax-funded healthcare.^18^ Patients with UC in Sweden are treated by gastroenterologists in specialized (non–primary care) outpatient clinics or hospitals.^19^

### Study Participants

Eligible participants were adults (≥18 years old) with ≥2 UC diagnostic listings according to International Classification of Diseases (ICD) codes (Appendix Table S2) recorded in the National Patient Register (NPR; nationwide coverage since 1987) (positive predictive value for IBD = 93%^20^^,^^21^) who had lived in Sweden for at least 12 consecutive months between January 1, 2006, and December 31, 2022. For the incident cohort, we included patients with a first UC diagnostic listing between January 1, 2008, and December 31, 2020.

Up to 10 reference individuals per patient with UC were randomly -selected from the general population. We individually-matched the references to participants with UC on the index date (date of first diagnostic listing of UC) by sex, birth year, and place of residence (at index date). Reference individuals did not have an IBD diagnosis before the first UC diagnosis of their matched UC participant. Reference individuals were censored if they were later diagnosed with IBD.

### Observation Period

The observation period for all study participants started on January 1, 2006, their 18th birthday, or the date when they were first diagnosed with UC (or the corresponding matching/index date for reference individuals), whichever came last. Only patients were diagnosed, while reference participants were assigned an index date. The observation period ended at death, emigration, or the end of the study period on December 31, 2022, whichever came first. In the incident cohort, the study period included a two-years observation period before the first UC diagnosis^22^ and at least two years after first diagnosis. In the analysis of the incident cohort, we determined the history of opioid use within two years of observation before the index date and therefore excluded participants with shorter observation times. In the prevalent cohort, all patients with a prevalent UC diagnosis during 2006-2022 and the reference individuals were required to reside in Sweden by the end of each observation year to be included in the estimate of that year.

### Data Sources

Using the Swedish personal identity number^23^ that is assigned to all residents, we linked data from several registers to our study population. We obtained individual-level data on vital status and place of residency from the Total Population Register^24^ and on education from the longitudinal integrated database for health insurance and labor market studies.^25^ Data on medical history were retrieved from the NPR,^26^ the Prescribed Drug Register (PDR; captures all pharmacy-dispensed medications in Sweden since 2005),^22^ and the National Cancer Register (nationwide coverage since 1958)^27^ (Appendix Table S3). In addition to the NPR and PDR, intravenous IBD treatments were also captured in the Swedish Inflammatory Bowel Disease Register (SWIBREG), which was established in 2005 to monitor and improve healthcare for patients with IBD.^19^ We used the Total Population Register^24^ to identify reference individuals without IBD as reference individuals.

### Variables and Definitions

#### Opioids

We received data on dispensed prescriptions of opioids as recorded in the PDR using Anatomical Therapeutic Chemical (ATC) codes (Appendix Table S4). The PDR captures all dispensed prescriptions from any medical doctor, regardless of where and by whom the doctor is employed. Based on oral morphine equivalents (Appendix Table S5), we categorized opioids as weak (with low-potency, eg, codeine combinations and tramadol) or strong (with high-potency, eg, oxycodone and morphine) as presented in the Appendix Table S6.

To look at participant use of opioids prior to first UC diagnosis, we classified participants as nonusers (no dispensation), intermittent users (≥1 dispensation in one-two periods), and persistent users (≥1 dispensation in all four periods) based on the number of six-month periods with a dispensation with two years (four 6-month periods) before first UC diagnosis. We defined chronic opioid use based on a two-year lookback period^28^ to account for potential changes in opioid use related to the preclinical phase and potential diagnostic delays in IBD.

### Mean Daily Dose Calculation

The mean daily dose (MDD) was derived by multiplying the dispensed dose (mg) by units (pills/patches) and the oral morphine equivalent conversion factor (Appendix Table S6). We then summed up the doses across each period and divided by the total number of days for all individuals in the same group and during the respective period: 365 days for the annual estimation and 365/2 days for the six-month periods. The formula is detailed in Appendix Table S4. Data on opioids administered (without a prescription) during hospitalizations were not possible to capture and were not included in this study.

### Comorbidity

Data on all cancer diagnoses (Chapter C from ICD10) and all psychiatric disorders (Chapter F from ICD10) as well as dispensed antidepressants (ATC: N06A) and anxiolytics (ATC: N05B) were obtained for all participants in our nationwide registers between 2006 and 2022 (Appendix Table S5).

### Statistical Analysis

For the incident cohort, we counted dispensed opioids and estimated the maximum daily dose (MDD) of opioids per 6-month interval per participant from 24 months prior to the first UC diagnosis until the end of observation among patients with UC and the corresponding time among reference individuals. For the prevalent cohort, we estimated the prevalence of ≥1 opioid dispensation and MDD of opioids per year, along with 95% CIs, among both the patients with UC and their matched reference individuals. We used Joinpoint software (version 5.0.2) to visualize the annual percentage change (APC) of opioid dispensation and to test for parallelism between participants with UC and reference individuals during 2006-2022.^29^ For assessing the annual trend of opioid use, we estimated the proportion of participants who used opioid during each year. We then visualized the annual percent change in opioid use for patients with UC and reference individuals and tested for parallelism. Patients with a prevalent UC diagnosis and reference individuals were ≥18 years old at the start of observation and alive by the end of each observation year. Patients with UC had two diagnoses before the start of observation. The reference patients had to be IBD free throughout the whole year. Finally, we excluded/terminated observation of patients with UC who lacked a matched reference patient and reference patients who did not have a matched patient with UC. The statistical analyses to estimate prevalence of opioid dispensation and MDD were performed using R version 4.4.0.

## Results

### Characteristics of the Study Participants: Incident Cohort

In the incident cohort, a total of 25 417 adults diagnosed with UC (2008-2020), and 253 405 matched IBD-free reference individuals were included (Table 1). Nearly half of the participants in both groups were female (48%-49%) and younger than 40 years at diagnosis (48%). Education levels were similar, with 17%-18% having ≤9 years of education and one-third (35%-36%) having education beyond high school. At baseline, history of cancer and psychiatric disorders and use of antidepressants or anxiolytics were similar, albeit numerically higher, among patients with UC evaluated during the two years before the first diagnosis. Among UC patients, nearly one-fourth (27%) had ulcerative proctitis (E1), and 24% and 20% had left-sided colitis (E2) and extensive colitis (E3), respectively.

**Table 1.** Characteristics of patients with incident adult-onset ulcerative colitis diagnosed 2008-2020 in Sweden.

Patient Characteristics	UC, No. 25 417	Matched reference individuals, No. 253 405
**Sex**		
** Men**	13 092 (52%)	130 489 (51%)
** Women**	12 325 (48%)	122 916 (49%)
**Year of first UC diagnosis**		
** 2008-2011**	6 052 (24%)	60 364 (24%)
** 2012-2015**	5 756 (23%)	57 401 (23%)
** 2016-2020**	9 751 (38%)	97 60 (38%)
**Age at first UC diagnosis, y**		
** 18-39**	12 239 (48%)	121 354 (48%)
** 40-59**	6 774 (27%)	67 605 (27%)
** ≥60**	6 404 (25%)	63 656 (25%)
**Education, y^a^**		
** ≤9**	4 325 (17%)	44 740 (18%)
** 10-12**	11 921 (47%)	113 364 (45%)
** >12**	8 789 (35%)	90 167 (36%)
** Missing**	382 (2%)	5 134 (2%)
**Medical history^b^**		
** Cancer^c^**	403 (2%)	2 471 (1%)
** Psychiatric disorders^d^**	1 696 (7%)	15 806 (6%)
** Antidepressants^e^**	3 684 (14%)	29 644 (12%)
** Anxiolytics^e^**	2 556 (10%)	20 191 (8%)
** Antidepressants or anxiolytics^e^**	4 873 (19%)	38 940 (15%)
**Ulcerative colitis extent^f^**		
** E1(ulcerative proctitis)**	7 026 (28%)	-
** E2 (left-sided colitis)**	6 124 (24%)	-
** E3 (extensive colitis)**	5 193 (20%)	
** EX (extent not defined)**	7 074 (28%)	-
**IBD treatment^g^**		
** Systemic corticosteroids**	17 718 (70%)	-
** Local corticosteroids**	6 627 (26%)	-
** Systemic 5ASA**	19 800 (78%)	-
** Rectal 5ASA**	16 421 (65%)	-
** Immunomodulators**	7 717 (30%)	-
** Targeted therapies**	5 500 (22%)	-
**Opioid use (within 2 years before the date of first UC diagnosis)^h^**		
** Nonuser**	21 173 (83%)	222 914 (88%)
** Intermittent user**	3 672 (14%)	26 511 (10%)
** Persistent user**	572 (2%)	3 980 (2%)

Abbreviations: UC, ulcerative colitis; 5ASA, 5-aminosalicylic acid.

a Highest recorded education level in LISA (from 1990 to the year of the 1st UC diagnosis).

b Within two years before the date of the first UC diagnosis or matching date.

c Source: National Cancer Register (within two years before the first UC diagnosis or matching date).

d Source: National Patient Register (within two years before the first UC diagnosis or matching date).

e Source: Prescribed Drug Register (from 2006 to the end of observation).

f Source: Swedish Inflammatory Bowel Disease register (SWIBREG).

g Source: National Patient Register, the Prescribed Drug Register and SWIBREG. Measured from the date of the first UC diagnosis to December 31, 2022.

h Nonusers did not receive opioids, intermittent users received ≥1 opioid dispensation for ¾ 6-month periods, and persistent users received ≥1 opioid dispensation all four 6 month-periods.

### Opioid use Around First UC Diagnosis Date: Incident Cohort

**Figure 1.**
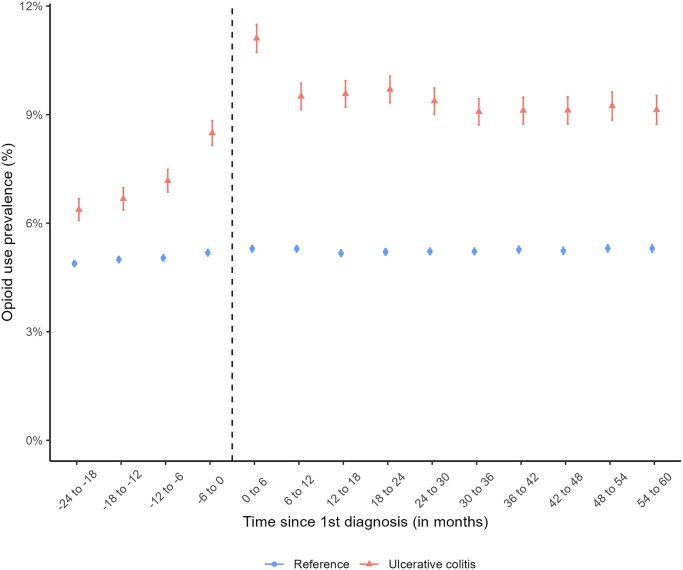
Opioid use prevalence among incident ulcerative colitis (UC) patients diagnosed 2008-2020 (weighted for sex and age at diagnosis date) and matched reference individuals based on six-month periods from two years before up to five years after first UC diagnosis in Sweden 2006-2022.

**Figure 2.**
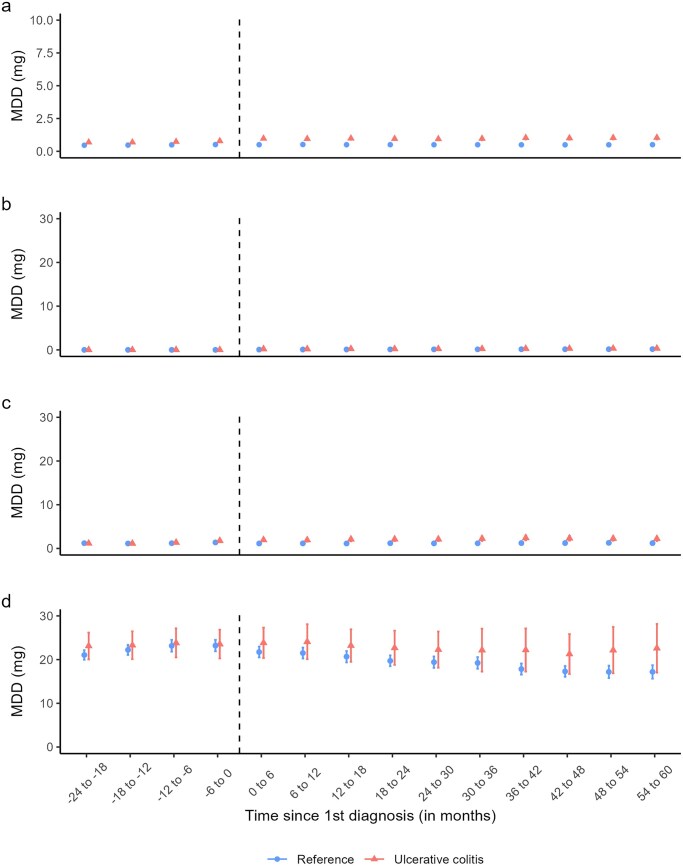
Mean daily dose (MDD) of dispensed opioids in milligram (mg) among participants with an incident ulcerative colitis (UC) diagnosed 2008-2020 and matched reference individuals during six-month periods from two years before and up to five years after the first UC diagnosis in Sweden 2006-2022. Panel a represent the overall study population, and panels b, c, and d present non-users, intermediate users and persistent users, respectively. We defined history of opioid use within four six-month periods (two years) before first UC diagnosis as Nonusers: no dispensed opioid; Intermittent users: ≥1 dispensed opioid in one to three of the six-month periods; and Persistent users, ≥1 dispensed opioid in all four six-month periods.

As presented in Figure 1, opioid use was already 1.3-fold higher among patients 18-24 months before first UC diagnosis (6.4% [95% CI, 6.1-6.7] compared with reference individuals (4.9% % [95% CI, 4.8-5.0]). Opioid use peaked during the one-six months after diagnosis among patients with UC (11.1%;[95% CI, 10.7-11.5) and stabilized at approximately 1.7-fold compared with reference individuals three-five years following the first UC diagnosis. A similar pattern was observed for strong and weak opioids (Appendix Figure S1). After stratifying based on the history of opioid use (no use, intermittent use, and persistent use) within two years before first UC diagnosis or match date (for reference individuals), opioid use following UC diagnosis or matching date was similar between patients with UC and reference individuals (Appendix Figure S2). The MDDs of opioids per six-month periods overall and stratified by opioid use history within two years of first diagnosis/matching date are presented in Figure 2. The MDDs of opioids among the overall UC population increased from approximately 0.69 mg (95% CI, 0.60-0.77) 19-24 months before diagnosis to 0.97 mg (95% CI, 0.87-1.07) at one-six months and 1.04 mg (95%, 0.89-1.19) in 55-60 months following diagnosis (Panel a).

### Annual Trend of Opioid Use Among Prevalent UC Patients: Prevalent Cohort

**Figure 3.**
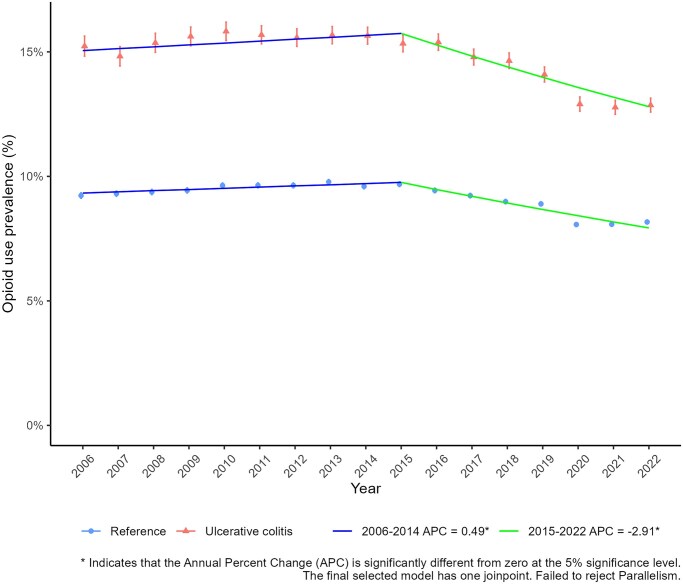
Joinpoint analysis of the annual prevalence of having ≥1 opioid dispensation among adults with a prevalent ulcerative colitis (UC) diagnosis and reference individuals (matched on sex, birth year, year of diagnosis, and residential location at diagnosis year) 2006-2022, Sweden.

**Figure 4.**
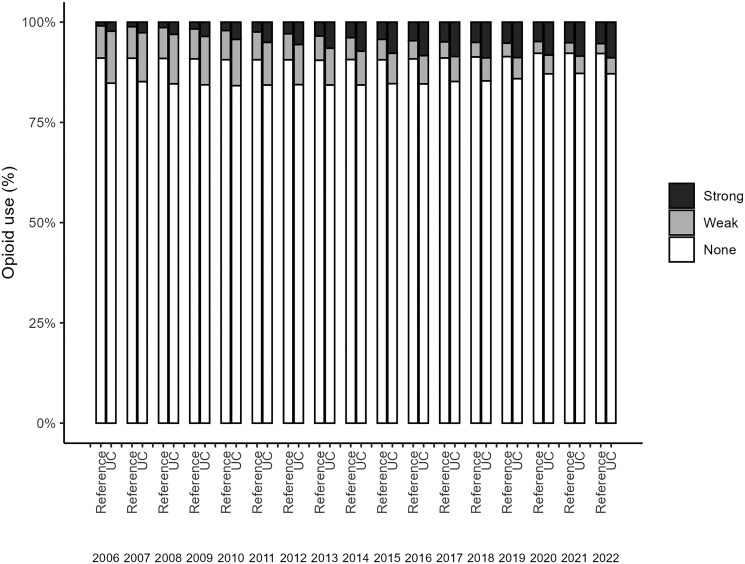
Annual proportion of adults with ulcerative colitis (UC) and reference individuals (matched on sex, birthyear, and residential location) with ≥1 filled strong or weak opioid dispensation 2006-2022, Sweden.

In the prevalent cohort, a total of 66 929 adults with prevalent UC at some point during 2006-2022 and 641 609 matched reference individuals were included (Table 2). The annual prevalence of opioid use among adults with UC changed from 15.2% (95% CI, 14.8-15.6) in 2006 to 12.9% (95% CI, 12.6-13.1) in 2022, a 15% reduction. In the same period among references, the prevalence of opioid use changed from 9.2% to 8.2% (11% reduction). As shown in Figure 3, both patients with UC and reference individuals exhibited a slight increase in annual opioid use during 2006-2015 (APC, 0.49), followed by a moderate decline during 2016-2022 (APC, −2.9). Stratifying based on strength of opioids showed that the use of stronger opioids increased during the observation period among both participants with UC and reference individuals while the use of weak opioids decreased in both groups ­(Figure 4). Among participants with UC, use of strong opioids increased from 2.3% in 2006 to 8.9% in 2022. Similarly, among reference individuals, use of strong opioids increased from 0.9% to 5.4% between 2006 and 2022.

**Table 2.** Characteristics of patients with a prevalent ulcerative colitis (UC) diagnosis in Sweden 2006-2022.

Patient Characteristics	UC, No. 66 929	Matched reference individuals, No. 641 609
**Sex**		
** Men**	35 309 (53%)	336 847 (53%)
** Women**	31 620 (47%)	304 762 (47%)
**Year of first UC diagnosis**		
** <1987**	6 704 (10%)	56 379 (9%)
** 1987-1997**	8 144 (12%)	72 834 (11%)
** 1998-2005**	17 676 (26%)	169 514 (26%)
** 2006-2010**	10 788 (16%)	107 532 (17%)
** 2011-2015**	10 249 (15%)	102 171 (16%)
** 2016-2022**	13 368 (20%)	133 179 (21%)
**Age at first UC diagnosis, year**		
** <18**	5 147 (8%)	49 496 (8%)
** 18-39**	30 668 (46%)	296 937 (46%)
** 40-59**	18 764 (28%)	179 265 (28%)
** ≥0**	12 350 (18%)	115 911 (18%)
**Highest completed education (years)^a^**		
** ≤9**	12 467 (19%)	118 562 (18%)
** 10-12**	29 121 (44%)	276 779 (43%)
** >12**	24 864 (37%)	239 583 (37%)
** Missing**	477 (1%)	6 685 (1%)
**Comorbidities^b^**		
** Cancer^c^**	760 (1%)	4 343 (1%)
** Psychiatric disorder^d^**	2 584 (4%)	24 411 (4%)
** Antidepressants^e^**	4 657 (7%)	38 267 (6%)
** Anxiolytics^e^**	3 189 (5%)	25 302 (4%)
** Antidepressants or anxiolytics^e^**	6 167 (9%)	49 963 (8%)
**Ulcerative colitis extent^f^**		
** E1**	9 387 (14%)	NA
** E2**	14 891 (22%)	NA
** E3**	30 290 (45%)	NA
** EX**	12 361 (18%)	NA
**IBD treatment during observation^g^ ^,^ ^h^**		
** Systemic corticosteroids**	45 420 (68%)	-
** Local corticosteroids**	16 356 (24%)	NA
** Systemic 5ASA**	47 352 (71%)	NA
** Rectal 5ASA**	34 574 (52%)	NA
** Immunomodulators**	19 055 (28%)	NA
** Targeted therapies**	11 347 (17%)	NA

Abbreviations: IBD, inflammatory bowel disease; NA, not applicable.

a The highest recorded education level in the longitudinal integrated database for health insurance and labour market studies (LISA) (from 1990 to the year of the 1st UC diagnosis).

b Within 2 years before the date of the first UC diagnosis.

c The National Cancer Register 1958-2020.

d The National Patient Register.

e The Prescribed Drug Register.

f None—users did not receive any opioids, intermittent users received opioids at least once, and persistent users received opioids every 6 months.

g A person is censored if he/she dies, emigrates or receives an IBD diagnosis.

h The National Patient Register, the Prescribed Drug Register, and the Swedish Inflammatory Bowel Disease register (SWIBREG).

The MDD trend among the prevalent UC population declined between 2006 and 2022 among both patients and the reference population (Appendix Figure S3). However, the decline was sharper among patients and amounted to approximately 67% of the initial use by the end of the observation period. The MDD per patient with UC was 1.5 mg in 2006 compared with 1.0 in 2022 while the corresponding MDDs for references were 0.75 and 0.55 mg, respectively.

## Discussion

### Main Findings

In this nationwide population-based register study, we investigated the use of prescribed opioids among adults with UC in Sweden compared with that of matched reference individuals from the general population. Among patients with UC, opioid use gradually increased during the two years preceding the first UC diagnosis, peaked within one-six months after diagnosis, and then stabilized three-five years postdiagnosis at a level higher than that of reference individuals. We also found that the prevalence of opioid use and the MDD of opioids had declined among patients with UC as well as the general population in Sweden between 2006 and 2022. These results provide an important foundation for healthcare decision-makers, clinicians, and patients with UC by clarifying overall opioid use and long-term trends, thereby supporting improved pain management strategies and more informed healthcare resource allocation.

### Comparison With Previous Studies

Most of the evidence on the use of opioids among adults with UC is based on studies performed in the United States, where a high rate of prescription of opioids in the past few decades has created an opioid use epidemic, with associated mortality and diminishing quality of life.^30^ Besides the study by Burr et al. using a primary care database from the United Kingdom, our study is to our knowledge the only large study conducted outside North America to report on use of opioids among patients with UC. Similar to our observation among patients with CD in Sweden,^28^ according to the present study the use of dispensed prescribed opioids was already higher among patients with UC than the reference individuals two years before their first diagnosis. Compared to results from the Burr et al. study, which found that one-third of the United Kingdom patients received opioids in primary care settings,^9^ the Swedish population received fewer opioids. Our definition of opioid use was similar to that of the Burr et al. study.^9^ During the overlapping observation period (2010-2013) Swedish patients with UC had an annual prevalence of opioid use of approximately 15% compared with 30% among patients with IBD in the Burr et al. study.^9^ The study by Lin et al. from the United States also reported 38% opioid use the year following diagnosis.^13^ Our estimates report on 6-month use and could not be directly compared with the Lin et al. estimate. However, the annual estimate of opioid use obtained for the prevalent cohort indicates that the use of opioids among Swedish adults with UC is lower compared to those in the United States.^13^

Two previous studies have examined the use of opioids among patients with prevalent UC. In the study by Abdalla et al. using data from the Crohn’s and Colitis Foundation of America (CCFA) Partners cohort, approximately 5% of the 1 872 patients with a UC/IC diagnosis (2011-2014) reported use of opioids.^16^ Colombel et al. (2017), in assessing the safety of vedolizumab across 30 countries (2009-2013), found that among 1 114 patients with UC, approximately 9% used opioids at study baseline.^31^

Three studies have focused on heavy/chronic (persistent) opioid use, of which two were from the United States (based on Market Scan databases) and one from Canada.^8^^,^^14^^,^^32^ In the Canadian study performed in 2014, Targownik et al. investigated the likelihood of becoming a “heavy” opioid user (received daily >50 mg opioids over 30 days at any 365-day window in ≥2 separate dispensations), and opioid-associated mortality following diagnosis among patients with IBD in Manitoba.^8^ In the Targownik et al. cohort, approximately 1 in 10 patients with IBD (11.0%) became heavy opioid users within 10 years after diagnosis; a 3-fold risk compared with matched reference individuals from the general population. Among the remaining studies from the United States, Noureldin *et al.* 2019 defined persistent opioid use as use of opioids for ≥90 days per year following IBD flare.^14^ According to this definition, nearly 1 in 3 patients was a persistent opioid user. And finally, Wren et al. used data from hospital visits in the Market Scan database and defined persistent opioid use as having ≥3 separate opioid drug claims on distinct dates within a 2-year rolling window, or ≥2 separate opioid drug claims on distinct dates within one year.^32^ The authors found that 1 in 3 patients had chronic opioid use and 1 in 4 patients had persistent chronic opioid use defined as lasting ≥4 years.

Compared to previous studies, our study had a longer observation period following diagnosis (up to five years) and also analyzed the two years prior to UC diagnosis. Previously, only Lin et al. have reported on the use of opioids in the last year before diagnosis and also included general population reference individuals.^13^ A main challenge in comparing the results across studies is the varying definition of opioid use and the choice of different time intervals. For example, heavy opioid use was defined based on the oral morphine equivalent and number of days with opioids in the Targownik et al.^8^ study compared with ≥4 prescriptions per year in the Burr et al. study,^9^ and ≥1 dispensed opioid per six-month periods during two subsequent years in the current study.

### Mechanisms and Clinical Implications

Although the prevalence of opioid use in our patient population was not substantially higher than in other settings, due to the risk of negative outcomes including increased morbidity, healthcare utilization,^33^ and mortality,^9^ opioid prescription should be limited to cases for which opioids are medically necessary and following careful discussions with patients about potential risks. In a nationwide register-based study, Kendler et al. investigated the effect of first opioid dispensation on being diagnosed with opioid use disorders among Swedish residents.^34^ This study found that among those with an opioid dispensation, 1% became opioid dependent, corresponding to a six-fold RR compared with patients without opioid dispensation. Patients with UC are less prone to persistent opioid use following IBD flares compared to patients with CD.^14^ Although the rate of becoming a persistent opioid user in the general population seems to be low, the comorbid psychiatric disorders and chronic pain and discomfort among adult IBD patients can increase their vulnerability to opioid dependence. However, a recent study from Manitoba, Canada, found no excess use of opioids among patients with IBD and comorbid psychiatric disorders, despite the association observed in the general population.^35^ This finding may reflect a high adherence to pain management guidelines and awareness among clinicians of the risks of opioid prescription among high-risk IBD patients (including those with psychiatric disorders).

In our study, we observed an increasing trend for using stronger opioids accompanied by an overall decline in the prevalence of opioid use and MDDs of opioids among both patients with UC and reference individuals. This decline in prescribed opioid use in Sweden may be explained by the national strategy to decrease prescription of weak opioids, eg, tramadol and potential legal consequences for malpractice in the country, which were initiated several years ago following a reportedly increased mortality risk linked to weak opioids. Another explanatory factor could be the introduction of targeted therapies and their impact on patient outcomes, including abdominal pain, thereby decreasing the need for analgesics, including opioids. This overall declining MDD trend may suggest a shift toward safer and more sustainable approaches to pain management in Sweden. For patients with UC, a further reduction in opioid use is desirable and can minimize the risk of long-term opioid dependence and related adverse effects. There seems to be a need to promote awareness and access to alternative pain management strategies and also inform clinicians and patients about opioid-related risks among healthcare providers in Sweden to further decrease opioid prescriptions among patients with UC. In summary, our study results set a foundation to understand the extent and trend of opioid use among patients with UC in Sweden in order to tailor pain management strategies that are more compatible with IBD treatment and have fewer adverse outcomes than opioids in this specific population.

### Strengths and Iimitations

The main strengths of this study were the inclusion of a nationwide UC population, a large general population reference cohort, and the use of prospectively recorded data from routine medical practice with high quality. Moreover, this study is to our knowledge the first nationwide longitudinal cohort study with non-IBD reference individuals as comparators to patients with UC. Only one of the previous studies had a nearly nationwide coverage using data from the United States National ­Hospital Ambulatory Medical Care Survey (NHAMCS).^36^ The current study has one of the longest observation times of opioid use among adults with UC. This study also has some limitations. The PDR (the source of data on dispensed opioids), lacks data on the actual consumption of opioids. Although using data on dispensed opioids might capture use of opioids more accurately than prescription data, some dispensed doses may not be used and we cannot rule out that study participants also used opioids that were not prescribed to them. In addition, we did not have data on opioid use during hospitalizations. Adults with IBD have a higher risk of receiving opioids during hospitalizations,^37^ which could lead to an underestimation of opioid use among patients with UC. Finally, we did not have data on the reason for, presence, or degree of pain, which makes it difficult to determine whether the opioid prescription was motivated by disease-related pain or other conditions with pain. Availability of data on for example, fecal calprotectin, C-reactive protein, or patient-reported pain data could have strengthened the analysis. However, the main aim of this ­primary investigation was to present the overall prescribed opioid use among patients with UC compared to general population.

In this nationwide Swedish study comparing approximately 67 000 adults with UC (including around 25 000 incident cases) with some 640 000 non‐IBD reference individuals, participants with UC had higher use of prescribed opioids during the two years before diagnosis. Their use of opioids further increased after diagnosis, remaining 1.7 times higher than among reference individuals from general population over three to five years. Overall, the use of prescribed opioids in Sweden is declining. Further awareness of consequences of opioid use and alternative pain management interventions may help to maintain this declining trend for adults with UC in Sweden.

## Supplementary Material

izaf278_Supplementary_Data

## Data Availability

Register-based data used on this research are not shared due to Swedish legislations.
