# Supplementary material for: Trends in Use of Prescribed Opioids in Incident and Prevalent Patients With Ulcerative Colitis: A Nationwide Study in Sweden
Source: Inflamm Bowel Dis. 2025 Dec 11;32(4):677–87. doi: 10.1093/ibd/izaf278 (PMC13046046; doi:10.1093/ibd/izaf278)
Supplement: izaf278_Supplementary_Data [file izaf278_supplementary_data.docx]

# **Trends in Use of Prescribed Opioids in Incident and Prevalent Patients with Ulcerative Colitis: A Nationwide Study in Sweden**

Mehdi Osooli, Siri Voghera, Gustaf Bruze, Caroline Nordenvall, SWIBREG Study Group§, Charlotte Hedin, Åsa H Everhov, Pär Myrelid, Jonas F Ludvigsson, Ola Olén

*§ Malin Olsson^a^, Henrik Hjortswang^b^, Jonas Bengtsson^c^, Hans Strid^d,e^, Marie Andersson^f^, Susanna Jäghult^g^, Jonas Halfvarson^b^, Martin Rejler^j,k^, Olof Grip^l^, Ulrika L Fagerberg^m,n^ , Karl Mårild^o,p^, Johann Hreinsson^q^, Pontus Karling^r^*

*^a^Department of Surgery, County Council of Östergötland and Department of Clinical and Experimental Medicine, Linköping University, Linköping Sweden; ^b^Department of Gastroenterology, County Council of Östergötland and Department of Clinical and Experimental Medicine, Linköping University, Linköping, Sweden; ^c^Department of Surgery, Sahlgrenska University Hospital/Östra, Gothenburg, Sweden; ^d^Department of medicine Solna, Karolinska Institutet, Stockholm, Sweden; ^e^Karolinska University Hospital, Stockholm, Sweden; ^f^Department of Internal Medicine, Södra Älvsborgs Hospital, Borås, Sweden; ^g^Department of clinical science and education, Södersjukhuset, Stockholm, Sweden; ^j^Department of Medicine, Höglandssjukhuset Eksjö, Region Jönköping County Council, Jönköping, Sweden; ^k^Jönköping Academy for Improvement of Health and Welfare, Jönköping University, Jönköping, Sweden; ^l^Department of Gastroenterology, Skåne University Hospital, Malmö, Sweden; ^m^Department of Women´s and Children´s Health, Karolinska Institutet, Stockholm ^n^Centre for Innovation, Research and Education, Region Västmanland, Västerås, Sweden; ^o^Department of Pediatrics, Institute of Clinical Sciences, Sahlgrenska Academy, Gothenburg, Sweden; ^p^Department of Gastroenterology, Queen Silvia Children’s Hospital, Gothenburg, Sweden; ^p^Department of Gastroenterology, Sahlgrenska University Hospital, Gothenburg, Sweden  ^r^Department of Public Health and Clinical Medicine, Umeå University, Umeå, Sweden.*

| **Table S1.** Prevalence of opioid use among adults with inflammatory bowel disease (IBD) or Ulcerative Colitis (UC) based on data from previous studies with a sample size of ≥1000 participants | | | | | | |
| --- | --- | --- | --- | --- | --- | --- |
| **Author, publication year** | **Country, setting** | **Data source** | **Number of**  **UC patients/ hospital visits** | **Study period** | **Opioid use prevalence**  **(95% CI)** | **Opioid use definition** |
| **Patients with an incident UC diagnosis** |  |  |  |  |  |  |
| Osooli *et al*. 2024 | Sweden,  nationwide | Swedish national patient register (NPR) | Adults with UC:  Matched references: | 2008-2021 | Incident UC:  6 months < DX:  6 months > DX: | Had ≥1 filled opioid prescription in six-months periods within two years before and up to five years following UC diagnosis |
| Lin *et al*. 2020 [1] | United States,  sub-national | IBM Watson Health Commercial Claims and Encounters Database | Patients with UC:  Matched reference: | 2007-2014 | Year 0:  Year 1:  Year 2: | Received at least one opioid prescription/injection during year 0 or 1 or 2 years after the index date |
| Burr *et al.* 2018 [2] | United Kingdom,  sub-national | The primary care database ResearchOne | 8,866 | 1990-2013 | 1990-1993:  1990-2013:  2010-2013: | Prescribed opioids at primary care settings  - None/infrequent use: <1 prescription/year  - Moderate use: 1–3 prescriptions /year  - Heavy opioid use: ≥4 prescriptions/year |
| **Patients with prevalent UC diagnosis** |  |  |  |  |  |  |
| Osooli *et al*. 2024 | Sweden,  nationwide | Swedish national patient register (NPR) | 32,306 | 2008-2021 | Prevalent UC  2008:  2021: | Had ≥1 filled opioid prescription per calendar year |
| Abdalla *et al.* 2017[3] | United States,  sub-national | Crohn’s and Colitis Foundation of America (CCFA) Partners cohort | 6,309 | 2011-2014 |  | Was on narcotics at the time of the internet-based survey |
| Colombel *et al*. 2017 [4] | International  (> 30 countries) | Six safety clinical trials on vedolizumab | 3,388 | 2009-2013 |  | On opioids at the time of study (baseline) |
| Limsrivilai *et al*. 2017 [5] | United States, single-centre | University of Michigan Hospital | 1,430 | 2013-2015 |  | Any opioid use during the study period |
| Lichtenstein *et al*. 2012 [6] | United States and Canada, multicentre | TREAT registry** | 6,273 | 1999-2010 |  | Any opioid use between enrolment and the six-month data collection period of the event or censoring |
| **Other studies** |  |  |  |  |  |  |
| Chhibba *et al.* 2021 [7] | United States, nationwide | US National Hospital Ambulatory Medical Care Survey (NHAMCS) | 785,000* | 2006-2017 | In the ED:  At discharge: | Received prescribed opioids during the visit at or when discharged from ED |
| Alley *et al*. 2019 [8] | United States, nationally representative | Marketscan databases | 76,171* | 2009-2010 |  | - No use, <30 days of use, 30–59 days of use, 60–89 days of use, and ≥90 days of use, based on the total number of days supplied in prescriptions in 2009 - Extended opioid use: ≥60 days’ supply of opioids in 2009 |
| Noureldin *et al*. 2019 [9] | United States, nationwide | Marketscan databases | 3,076 | 2009-2015 |  | Persistent opioid use 90‐365 days following the IBD (index) flare |
| Wren *et al*. 2018 [10] | United States, nationally representative | Marketscan databases | 93,668* | 2007-2015 |  | Had ≥3 separate opioid drug claims on distinct dates within a 2-y rolling window, or ≥2 separate opioid drug claims on distinct dates within 1 year |
| Pauly *et al*. 2017 [11] | US, nationally representative | Marketscan databases | 47,164* | 2009-2012 |  | Had at least a 90-day supply of opioids in a six-month period without any 30-day gaps between prescriptions |
| Li *et al*. 2016 [12] | United States,  single-center | Cleveland Clinic (tertiary referral center) | 1,331 | 1998-2014 |  | Received narcotics while hospitalized (excluding after colectomy) or at time of transfer to the hospital |
| Targownik *et al*. 2014 [13] | Canada, Manitoba | Manitoba IBD Epidemiology Database | 4,217 | 1996-2010 |  | Heavy opioid use: received daily >50mg morphine equivalent opioid over 30 days at any 365-day window and had received at least two separate dispensations in the same period. Heavy opioid use within 90 days before and after diagnosis was excluded. |
| Abbreviations: CI: confidence interval; DX: Diagnosis; ED: emergency department; inflammatory bowel disease (IBD); HR: hazard ratio; NR: Not reported; SIR: standardized Incidence rate ratio; PY: Person-years; TREAT: Observational Crohn’ s Therapy, Resource, Evaluation, and Assessment Tool; UC: Ulcerative colitis.  * Number of hospital visits | | | | | | |

| **Table S2**. International Classification of Disease (ICD) codes defining inflammatory bowel diseases (IBD) | | | | |
| --- | --- | --- | --- | --- |
|  | **ICD-7 (1964-1968)** | **ICD-8 (1969-1986)** | **ICD-9 (1987-1996)** | **ICD-10 (1997-)** |
| Crohn’s disease (CD) | 572,00;  572,09 | 563,00 | 555 | K50 |
| Ulcerative colitis (UC) | 572,20;  572,21;  578,03 | 569,04; 563,1; 563,10; 569,02 | 556 | K51 |
| IBD unclassified (IBD-U) | UC+CD | UC+CD  or:  563; 563,0; 563,9; 563,98; 563,99 | UC+CD | UC+CD,  or K52.3 |
| ICD codes were captured prospectively in routine medical practice in the National Patient Register, as in several previous reports[13, 14]. Universal access to publicly funded health care is available for all residents, independent of residence, socioeconomic status, and disease severity. Having ≥1 international classification of disease code (ICD) for CD  Because definitions of exposure should not “look into the future”, subtypes of IBD were defined in accordance with the first two diagnostic codes only (*i.e*., no information after start of follow-up contributed to the subtype definition) [3, 4, 15]. | | | | |

| **Table S3.** An overview of the data sources used in the study | | | |  |
| --- | --- | --- | --- | --- |
| **Register name** | **Administrator** | **Source data** | **Coverage** | |
| Total Population Register [6] | Statistics Sweden (SCB) | Data on all persons registered in Sweden, including birth date, sex, place of residence (parish, municipality, or county, depending on availability of reference individuals), last immigration, emigration dates and date of death. | 1968-2020 | |
| National Patient Register [10] | Swedish National Board of Health and Welfare | Data from outpatient and inpatient hospital visits, including date of visit, primary and secondary diagnoses and procedures | 1964-2020 (outpatient surgery since 1997 and outpatient visits since 2001) | |
| Prescribed Drug Register [5] | Swedish National Board of Health and Welfare | The PDR was established in July 2005 and contains all prescribed drugs dispensed at pharmacies in Sweden. | 2005-2020 (national coverage since 2006) | |
| Cancer Register [11] | Swedish National Board of Health and Welfare | The cancer register was established in 1958 and contains data on all cancer diagnoses in Sweden. | 1958-2020 | |
| Cause of Death Register [12] | Swedish National Board of Health and Welfare | Main and underlying causes of death | 1952-2020 | |

| **Table S4**. International Classification of Diseases (ICD) 10 codes and Anatomical Therapeutical Chemical (ATC)* classification system codes used to capture psychiatric disorders* and cancer comorbidities** | | |
| --- | --- | --- |
| **Condition or drug** | **ICD10** | **ATC** |
| Cancer | Chapter C | - |
| Psychiatric disorders | Chapter F | - |
| Antidepressants | - | N06A |
| Anxiolytics | - | N05B |
| * in the prescribed drug register (PDR)  ** in the National Patient Register (NPR)  *** in the cancer register | | |

| **Table S5.** Formulae used to convert doses for various opioid products to oral morphine equivalents (OME) |
| --- |
| Dose = Quantity (number of packages) × Size (number of tablets/suppositories/doses/bandaid per package) × Strength (mg per tablet/suppository/dose, or micrograms per hour for bandaid) × bandaid (1 if it is not a bandaid, otherwise number of hours that the bandaid should be on) × OME (Table S6) |

**Table S****6.** Included ATC-codes and conversion rates for opioids. Anatomical Therapeutic Chemical (ATC) Classification System codes starting with N02A or R05DA0, Name, Route of administration, Defined Daily Dose (DDD), equianalgesic dose ratios^1-4^ and classification as strong or weak opioid.

| **ATC code** | **Name** | **Route** | **DDD (mg)** | **Equianalgesic dose ratio** | **Strong (S) or weak (W) opioid** | |
| --- | --- | --- | --- | --- | --- | --- |
| 1. N02AA01 | Morphine | PO | 100 | 1 | S | |
| 1. N02AA03 | Hydromorphone | PO | 20 | 6 | S | |
| 1. N02AA05 | Oxicodone | PO | 75 | 1.5 | S | |
| 1. N02AA55 | Oxicodone combinations | PO | 75 | 1.5 | S | |
| 1. N02AB01 | Ketobemidone | PO | 50 | 1 | S | |
| 1. N02AB02 | Pethidine | PO | 400 | 0.1 | S | |
| 1. N02AB03 | Fentanyl | TD | 1.2 | 100 | S | |
| 1. N02AE | Buprenorphine | TD | 1.2 | 110 | S | |
| 1. N02AE | Buprenorphine | SL | 1.2 | 50 | S | |
| 1. N02AF | Nalbuphine |  |  | 0 filled prescriptions | S |  |
| 1. R05DA04 | Codeine |  |  | 0 filled prescriptions | W | |
| 1. N02AX02 | Tramadol | PO | 300 | 0.2 | W | |
| 1. N02AX06 | Tapentadol | PO | 400 | 0.4 | W | |
| 1. N02AC04 | Dextropropoxyphene (chloride) | PO | 200 | 0.1 | W | |
| 1. N02AC04 | Dextropropoxyphene (napsylate) | PO | 300 | 0.1 | W | |
| 1. N02AC54 | Dextropropoxifen combinations excluding psycholeptics | PO | 140 | 0.15 | W | |
| 1. N02AJ13 | Tramadol and paracetamol |  |  | 0 filled prescriptions | W | |
| 1. N02AJ06 | Codeine and paracetamol |  |  | 0 filled prescriptions | W | |
| 1. N02AJ08 | Codeine and ibuprofen |  |  | 0 filled prescriptions | W | |
| 1. N02AJ09 | Codeine and other non-opioid analgesics |  |  | 0 filled prescriptions | W | |
| 1. N02AA59 | Codeine combinations excluding psycholeptics | PO | 100 | 0.1 | W | |

PO = per oral, TD = transdermal, SL =sublingual, R = rectal

|  |
| --- |
|  |
|  |

| ^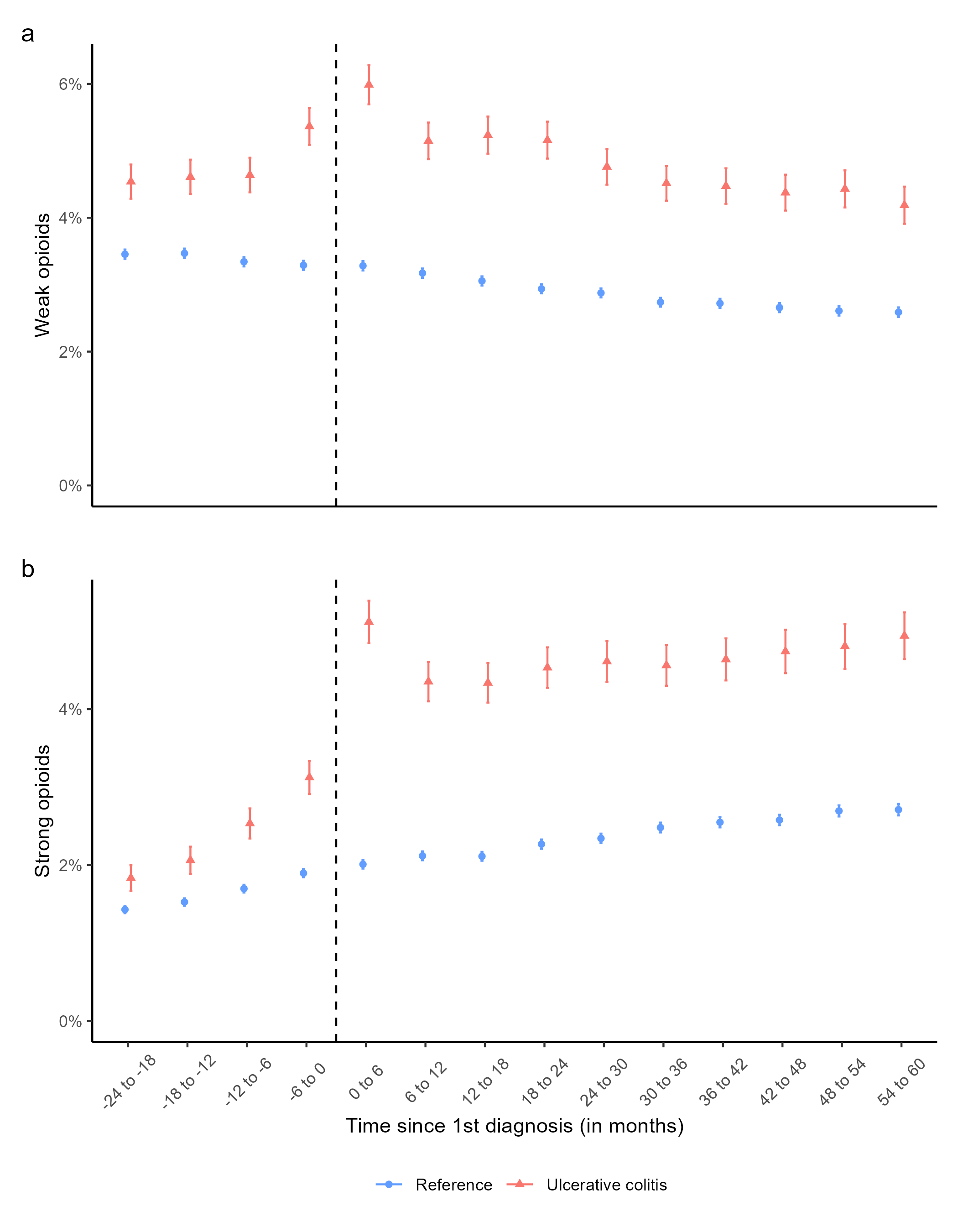^ |
| --- |
| **Figure S1.** Prevalence (weighted) of weak (upper panel) and strong (lower panel) opioids use among patients diagnosed with Ulcerative Colitis (UC) 2008-2019 and matched reference individuals based on six-month periods from two years before up to five years after first UC diagnosis. |

| 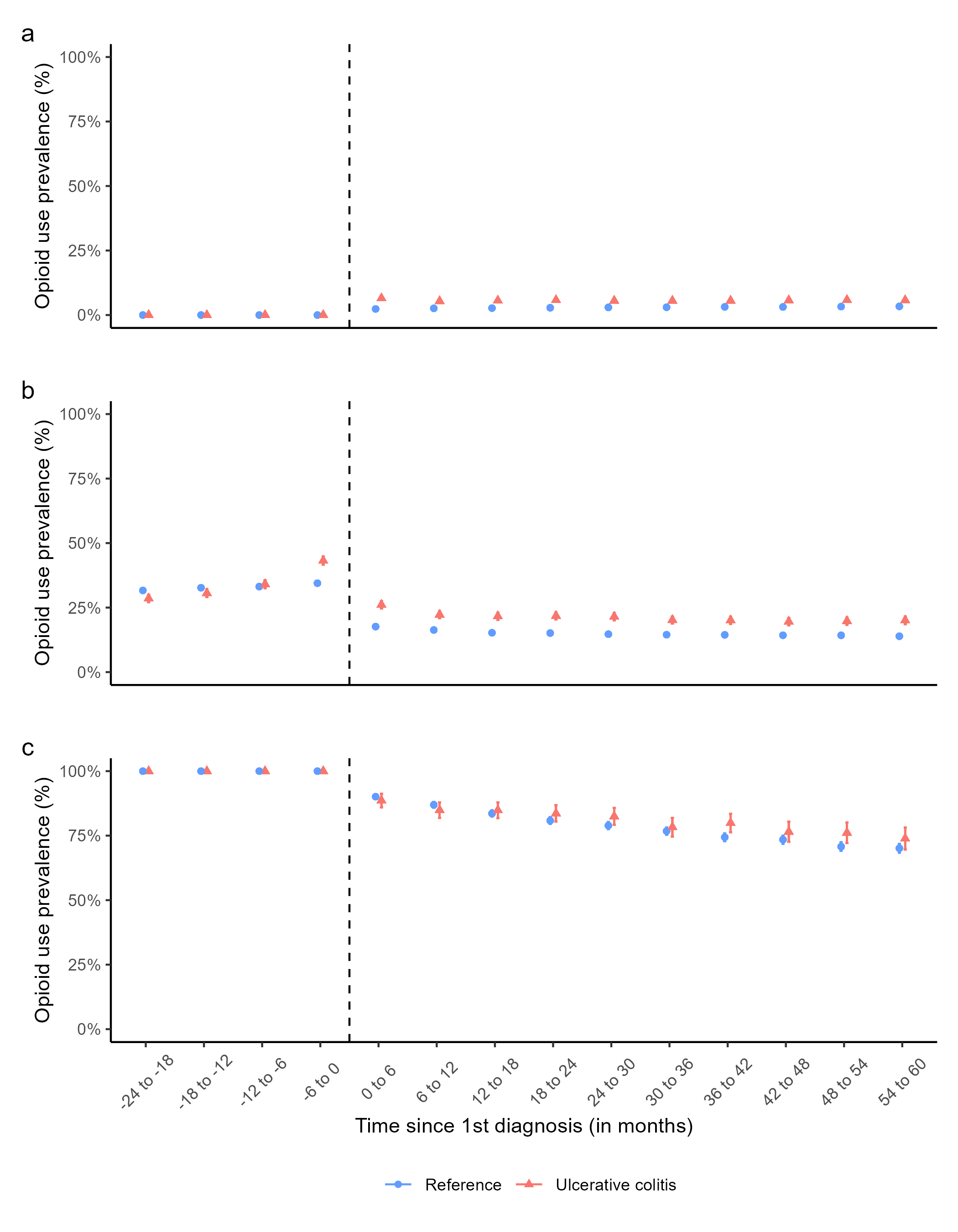 |
| --- |
| **Figure S2** Annual proportion of adults with Ulcerative Colitis (UC) and reference individuals (matched on sex, birthyear, and residential location) with ≥1 filled strong or weak opioid prescription 2006-2022, Sweden |

| 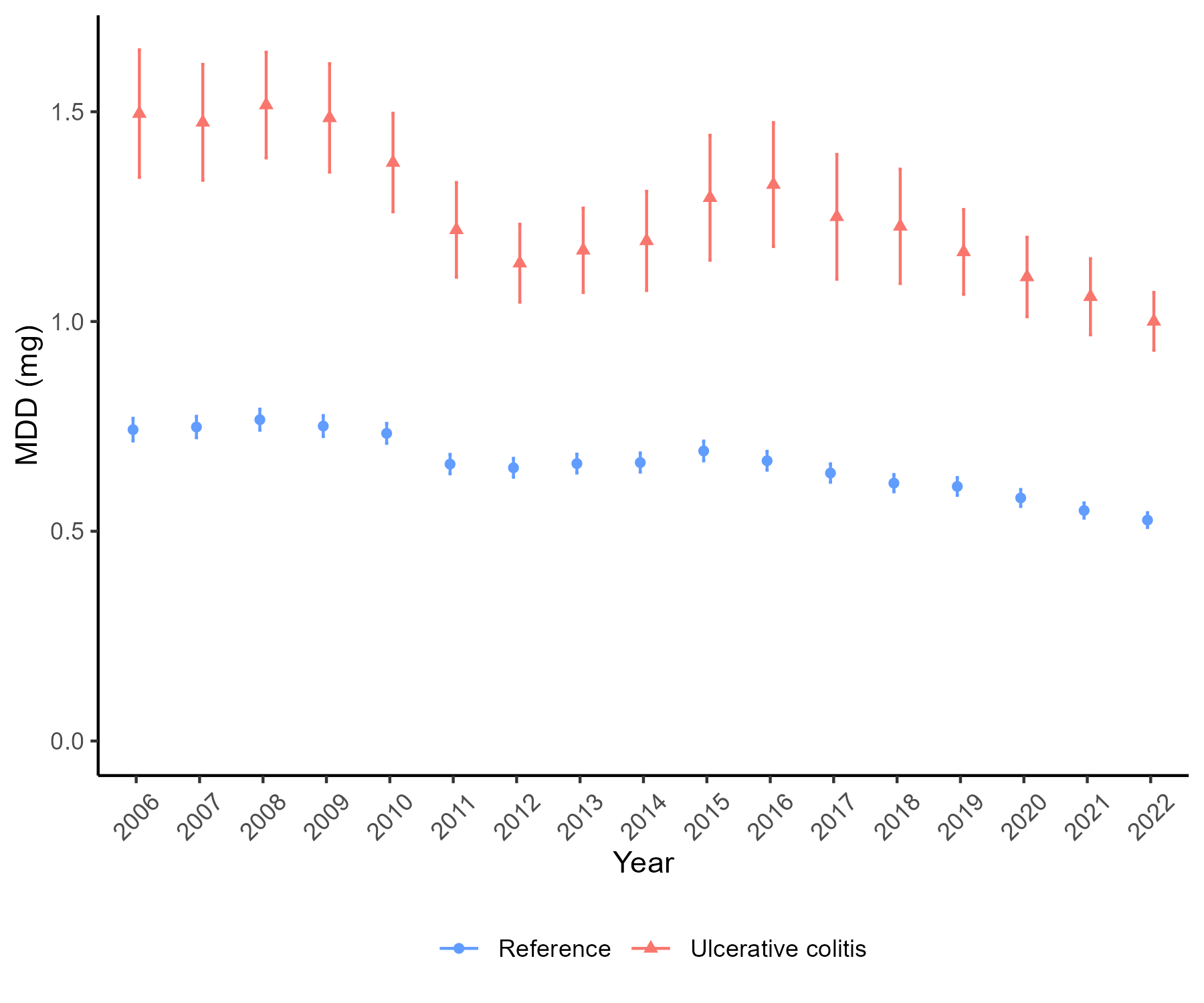 |
| --- |
| **Figure S3.** Mean daily dose (MDD) of opioids among patients with a prevalent ulcerative colitis (UC) diagnosis 2006-2022 and matched reference individuals (weighted) based on sex, birth year, and residential location at the year of matching. The MDD is calculated as the sum of the prescribed doses per “person and year” divided by 365 (number of days). |
